# Assessing stakeholder inclusion within high pathogenicity avian influenza risk governance strategies in the United Kingdom and United States

**DOI:** 10.3389/fvets.2025.1547628

**Published:** 2025-04-17

**Authors:** Kimberly Lyons, Darrell R. Kapczynski, Samantha J. Lycett, Paul Digard, Lisa Boden

**Affiliations:** ^1^Global Academy of Agriculture and Food Systems, Royal (Dick) School of Veterinary Studies, University of Edinburgh, Edinburgh, United Kingdom; ^2^Exotic and Emerging Avian Diseases Research Unit, U.S. National Poultry Research Center, Agricultural Research Service, USDA, Athens, GA, United States; ^3^Roslin Institute, University of Edinburgh, Edinburgh, United Kingdom; ^4^Royal (Dick) School of Veterinary Studies, University of Edinburgh, Edinburgh, United Kingdom

**Keywords:** risk governance, stakeholder, avian influenza, avian flu, stakeholder map

## Abstract

Since 2020, outbreaks of high pathogenicity avian influenza (HPAI) have led to a global rise in deaths of both wild birds and poultry, as well as an increase in reported cases of HPAI detected in mammals. These outbreaks have had negative impacts on poultry producers, trade, and wild bird populations. Risk governance frameworks for emerging infectious diseases such as HPAI encourage outbreak policies to be grounded in a variety of stakeholder perspectives and for there to be effective, transparent communication between all those involved. However, the COVID-19 pandemic exemplified how collaboration is not always easy to implement, leading to potentially sub-optimal outbreak response processes. To our best knowledge, there is limited to no current research assessing the stakeholder landscape and outbreak decision-making and response processes in the United Kingdom (UK) and United States of America (USA) for the recent HPAI outbreak. In this study, 20 key stakeholders involved in outbreak decision-making and response in the United Kingdom and United States were asked to provide their insights into the structure of stakeholder landscape, communication pathways, and challenges in decision-making and response implementation for their respective countries. Semi-structured interviews were conducted with participants from the United Kingdom and United States; participants included policy advisors, veterinarians, researchers, and poultry industry representatives all involved in HPAI outbreak processes in their country. From these interviews, stakeholder maps for all those involved in HPAI decision-making and response were created for the UK and USA. This study concluded that smallholders and backyard poultry owners need to be better represented in policy-industry communication pathways and that improved information sharing at the policy-science and policy-industry interfaces is essential to ensure an efficient outbreak response.

## 1 Introduction

Avian influenza (AI) is an influenza A virus that primarily infects domestic and wild birds (1). Influenza A viruses are classified according to the subtypes of their hemagglutinin (HA; H1-H18) and neuraminidase (NA; N1-N9) surface proteins, and of the 18 known HA subtypes, 16 of them are found in avian species (2). AI can be characterized by its severity: low pathogenicity avian influenza (LPAI), which generally produces minimal or no clinical signs in domestic poultry, or high pathogenicity avian influenza (HPAI), which produces severe clinical signs and high mortality in poultry (3, 4). To date, only subtypes H5 and H7 have been demonstrated to have highly pathogenic forms (2).

In 2020, the highly pathogenic variant virus of HPAI first detected in China in the late 1990s and responsible for global outbreaks since 2014 (1), subtype H5Nx clade 2.3.4.4b, began spreading in Africa, Asia, and Europe, causing unprecedented mortality rates in wild birds and poultry (5–7). In 2021, this H5N1 variant spread from Europe to North America (5) and in 2022, to South America (8). This most recent circulating strain has spilled over into mammals, including farmed mink, sea lions, domestic cats, and more recently, dairy cattle and swine (9–14); the ability of the virus to infect other mammals poses a potential zoonotic threat to humans (15).

HPAI outbreaks are notifiable to the World Organization for Animal Health (WOAH), an organization comprised of 183 member countries founded on an international agreement to combat animal disease (16, 17). When HPAI is detected in poultry in a member country, that country is responsible for notifying WOAH and international trade is restricted until the country returns to disease-free status (18). As a result, many countries employ a stamping-out procedure, meaning that when HPAI is detected on a premises, poultry are culled to eradicate the disease (19). The carcasses will then be disposed of and premises undergo primary and secondary cleaning and disinfection to ensure they are disease-free before new poultry can be placed (18). Confirmed infection on premises and the subsequent flock depopulation can negatively impact farmers, the poultry industry, and trade (20).

Due to how quickly HPAI can spread within and to other premises, rapid detection and response is important (21). Roodenrijs et al. (22) emphasizes the time pressure that accompanies effective control of infectious diseases to prevent rapid spread, as decision-making needs to occur as quickly as possible following a suspected outbreak. Animal health policy decision-making is typically based on risk assessment, where risk communication often occurs linearly; the decision-makers receive risk assessments, develop policy, and then communicate their decisions to other stakeholders. This communication process does not always consider the complexity of the stakeholder landscape, which may reflect different views of risk and mitigation strategies, nor is it typically well-designed to receive feedback from relevant stakeholders who are not already directly involved in decision-making. Pfeiffer (23) comments that ineffective risk management policies have often resulted from a lack of communication between the different stakeholders involved in risk management.

Millstone et al. (24) proposed a more transparent model of the risk analysis framework, which suggests that risk governance policies should be grounded in the perspectives of a wide scope of stakeholders instead of a narrow scope that typically only includes policymakers (25). This approach encourages communication and collaboration between all stakeholders, including non-scientific ones, which in turn would assist with greater acceptance of policy decisions (24, 26).

It is important that stakeholders from all sectors, including policy, research, poultry and other livestock industries, and any other impacted sectors, take a collaborative approach to minimize the risk of incursion and disease spread and ensure established processes function as they should (21). As seen during COVID-19, there were variances in how the response was supposed to function and how it actually did (27). For example, despite using epidemiological models as a basis for policy decisions in the United Kingdom, due to gaps in knowledge and poor coordination between different implementing bodies, the employment of COVID-19 control measures was often delayed. Better collaboration between scientists and those carrying out disease response activities at the ground-level was needed to ensure effective response to outbreaks (28). It is therefore beneficial to gain insight from the stakeholders involved following an animal disease outbreak to determine if or where the outbreak response to HPAI did not function as it should and how it could be improved.

In countries such as the United Kingdom (UK), which has just experienced its largest outbreak in its history (29) and the United States of America (USA), which is one of the top three poultry meat producers in the world (30), early detection of disease, rapid decision-making, and effective disease outbreak response are vital to reducing the likelihood of further HPAI impacts. Recent studies examining the UK and USA response to HPAI during previous outbreaks have focused on how specific stakeholder groups can mitigate the risks of HPAI, such as smallholders and wildlife managers (31, 32). Despite greater need for stakeholder collaboration and communication for effective infectious disease decision-making and response (14), to our knowledge there is little to no current research mapping the stakeholder landscape and evaluating the impact of stakeholder communication pathways on risk governance in response to the most recent strain of HPAI in these two nations.

This study sought to examine the decision-making processes in place for a HPAI outbreak in the UK and USA to gain insight into how to better manage risk governance processes. This included a stakeholder mapping analysis to identify who is involved in HPAI risk governance, decision-making and response; what the stakeholder communication pathways looked like in the UK and USA; and, where collaboration exists or needs to be improved for improved risk governance during future outbreaks.

## 2 Materials and methods

### 2.1 Methodology

In this study, hour-long in-depth interviews were conducted with UK and USA participants involved in avian influenza outbreak preparedness and response from the government, research institutions, and poultry industry associations. The interview guide (Supplementary Table 1) was comprised of four sections with four discrete aims:

1. To gain an understanding of the participant and their role in avian influenza preparedness and response;
2. To identify the stakeholders involved in decision-making around avian influenza outbreaks, as well as the data and expertise needed to make decisions and any challenges that arose;
3. To identify the stakeholders involved in the implementation of outbreak response activities, the data and expertise needed to implement this response, and any challenges with this; and,
4. To explore data that would be useful to collect and use for predicting virus movement, warning premises to prevent potential outbreaks, and build into HPAI-related policies.

Participants were initially recruited by contacting known stakeholders within the professional networks of the lead researchers, and then subsequently identified using a snowball sampling approach to recruit further participants. Initial participants included those working with animal disease policy, including policy decisions around avian influenza outbreaks, those conducting scientific research on avian influenza virus, and poultry industry professionals who were in regular contact with broiler, egg, and turkey farmers. All interviews were conducted in English. Expertise refers to the knowledge and experience required for outbreak decision-making and response implementation.

### 2.2 Data collection

Prior to interview, participants were provided with an information document outlining the purpose of the project and asked to provide informed consent. Interviews were conducted by one of two researchers in English, via Microsoft Teams. With the permission of participants, the interviews were recorded and anonymised at the point of transcription. Following transcription, the recordings were deleted to preserve participant anonymity. The Human (Research) Ethical Review Committee at the Royal Dick School of Veterinary Studies and Easter Bush Campus approved this study (study reference number HERC 822_21).

The initial UK interviews were conducted in 2021 with 12 participants. In 2023, five of the participants who had agreed to be contacted in the future were recruited for a follow-up interview to determine if there were any changes to previous responses. Of the five contacted, three participated in a second interview, with a total of 15 UK interviews conducted with 12 participants. Eight individuals from the USA were interviewed in total in 2023.

The breakdown of participants in each country by their role is provided in Table 1.

**Table 1:** Number of participants in each role within each country interviewed/surveyed.

Number of participants					
Country	Policy officers/advisors	Veterinarians	Researchers	Industry representatives	Total
United Kingdom	3	3	4	2	12
United States	3	2	2	1	8
Total	6	5	6	3	20

### 2.3 Data analysis

Interview data was coded and analyzed using the software NVivo by Lumivero. Interview responses were first examined to produce a communication network map of decision-makers and response implementers during HPAI outbreaks in the UK and USA and to identify the flow of information between each group. The mapping process required coding for all decision-makers and response implementers identified by interview participants, which ones were ranked as most important or least important, and the different methods of data and information sharing that occurred between them. The nature of the study involves many different organizational and other acronyms, so a separate table of abbreviations is also supplied (Supplementary Table 2).

A thematic content analysis (33) was then used to code the data pertaining to the strengths and challenges experienced during HPAI preparedness, decision-making, and response implementation. Responses were split out according to the following themes: stakeholder collaboration, information and data sharing, rapid detection and response to outbreaks, resource availability, and disease prevention. Themes were created based on the questions in the questionnaire (Supplementary Table 1) and the frequency with which participants mentioned them across the two countries.

## 3 Results

Participants from the UK and USA provided details into the stakeholders involved in decision-making and response implementation in their country, the collaboration and information sharing that must occur between these different stakeholders, as well as areas to improve for better HPAI outbreak preparedness. Table 2 provides a summary of the challenges identified in HPAI outbreak response in the UK and USA and allows for a comparison across the themes of stakeholder collaboration, information and data sharing, rapid detection and response, resource availability, and disease prevention. Participants in both the UK and USA described some similar challenges across the themes, such as a need to better include smallholders and backyard owners in existing communication networks, carrying out rapid response on premises in remote locations, and the ability for poultry farms to take further preventative measures against HPAI due to cost. There were also numerous challenges that were specific to only the UK and/or USA. The identified challenges are expanded upon later in the results.

**Table 2:** Comparison of challenges experienced with HPAI outbreak decision-making and response implementation in the United Kingdom and United States.

Challenges in the HPAI outbreak response		
Theme	United Kingdom challenges	United States challenges
Stakeholder collaboration	Need to include smallholders and backyard owners in Avian Core Group. Distrust between government and smallholders and backyard owners.	Need to include smallholders and backyard owners in existing communication networks. Lengthy process to enact change disincentivises industry to feedback to policymakers. Distrust in CDC to handle zoonotic disease outbreaks. States may not trust other states to carry out proper HPAI-related procedures.
Information and data sharing	Scientists experience delays in government sharing data. Difficult user experience when searching for HPAI-related information on government website. National Poultry Register may have out-of-date or incomplete data. Need to minimize number of steps in communication between policymakers and poultry workers.	HPAI-related information on government websites may not be accessible or applicable for non-commercial farmers. Difficult to direct laypeople to appropriate government websites for information.
Rapid detection and response	Remote location of infected premises. Financial, emotional, and mental toll of HPAI outbreaks on bird owners.	Remote location of infected premises. Financial, emotional, and mental toll of HPAI outbreaks on bird owners. Impacts of climate on equipment usability. Winter conditions creating safety hazards for response team. Animal welfare concerns with depopulation methods.
Resource availability	Continual movement within government departments causes lack of or loss of expertise at the policy level. Lack of poultry-specific expertise in general veterinarians.	Burnout and lack of training time causes lack or loss of expertise at the policy level.
Disease prevention	Individual compliance with on-farm biosecurity may differ. Increased predictive capabilities of HPAI outbreaks may cause inconsistency in biosecurity application. Ability to undertake preventative measures against HPAI may be limited.	Individual compliance with on-farm biosecurity may differ. Increased predictive capabilities of HPAI outbreaks may cause inconsistency in biosecurity application. Ability to undertake preventative measures against HPAI may be limited.

### 3.1 Outbreak response processes

In order to understand the extent of the challenges identified through the interviews, participants first described the steps involved in declaring an HPAI outbreak and stamping out the disease on the infected premises, as well as the stakeholders involved in decision-making and response implementation.

**Figure 1:**
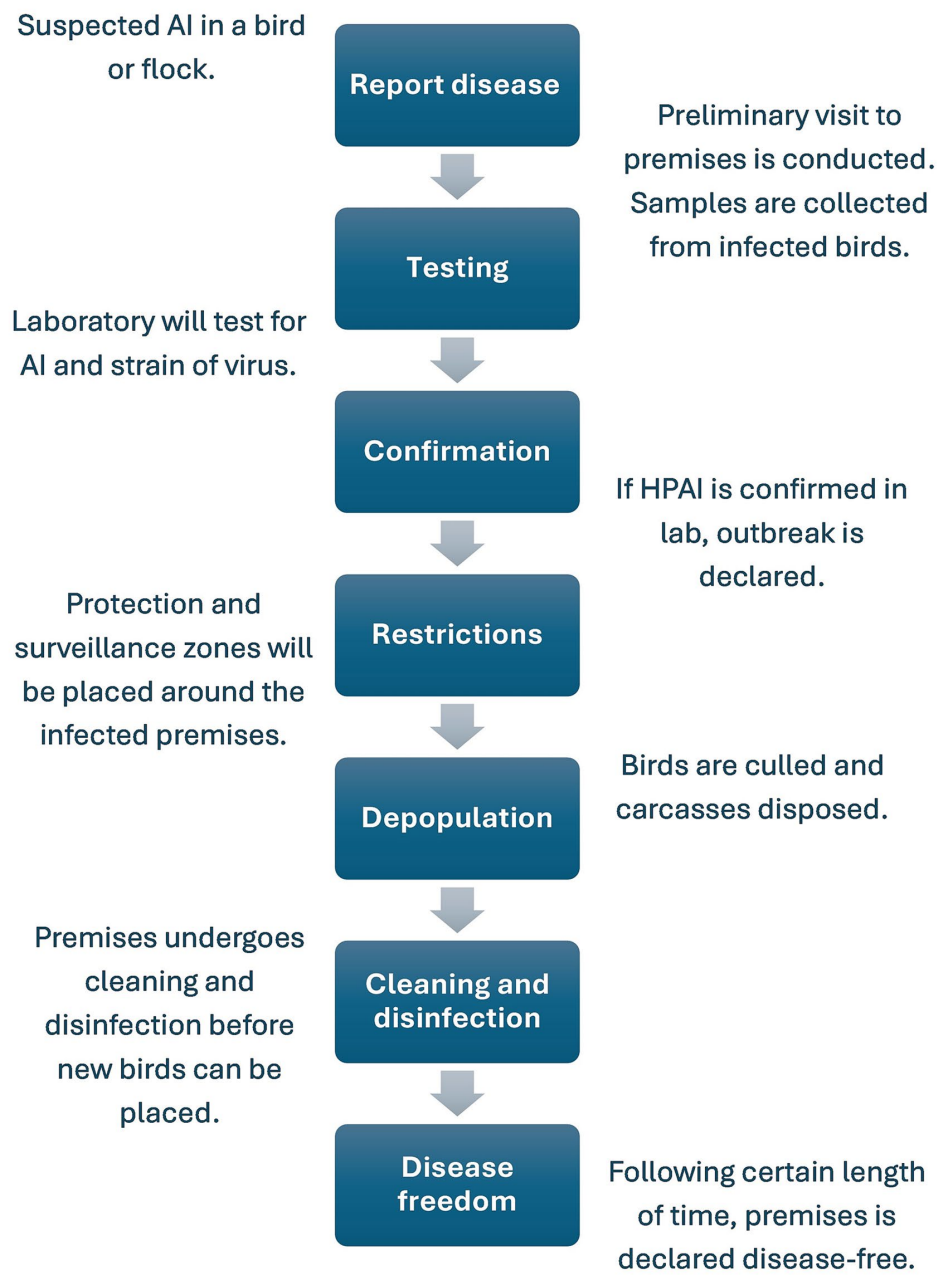
A high-level overview of the activities that take place from the time HPAI is suspected on a premises to the point that the premises can be declared disease-free.

Both the UK and USA have documents providing detailed guidelines for what steps need to occur once suspected HPAI is reported on a premises (34, 35). UK and USA participants corroborated these details and shared insight into which stakeholders were responsible for the actions taken at each step. An overview of the steps that take place during the response is shown in Figure 1.

#### 3.1.1 United Kingdom outbreak response processes

In the UK, HPAI on premises is typically detected through passive surveillance by poultry owners and veterinarians. Poultry owners and veterinarians are legally required to contact the Animal and Plant Health Agency (APHA), an agency that sits within the Department for Environment, Food and Rural Affairs (Defra) of the UK government, if they suspect HPAI. Following this, APHA will place temporary restrictions on the premises, such as increased biosecurity measures, the housing and isolation of birds, and movement restrictions on poultry and poultry products (36). APHA will send a veterinary inspector from the Veterinary Exotic Notifiable Disease Unit (VENDU) to attend the premises and check the birds for clinical signs of HPAI, as well as to collect samples, which will be sent to the UK’s national reference center for avian influenza, Weybridge Laboratory. This will either confirm that HPAI is not present, whereby the temporary restrictions would be lifted on the premises, or will confirm HPAI, in which case the Chief Veterinary Officer (CVO) of the affected country would declare an outbreak.

The UK is comprised of England and the devolved administrations of Scotland, Wales, and Northern Ireland. Each devolved administration has their own CVO. The CVO of England also acts as the CVO of the entire UK and when a premises tests positive for HPAI, while the CVO of the impacted devolved nation is the one to declare the outbreak, the CVO of the UK is responsible for notifying WOAH. The infected premises are then subjected to a protection and surveillance zone with a three- and 10-km radius, respectively, which can overlap when there are multiple infected premises. The protection zone is kept in place for 21 days and surveillance zone for 30 days. Additionally, when a premises is confirmed to have an H5N1 strain of HPAI, a restricted zone is also established for 30 days; the radius of this zone is decided by risk assessment (34).

Upon confirmation of HPAI on the infected premises, APHA will carry out on-farm depopulation and disposal of carcasses. The method of culling depends on the type of bird and size of the flock; cervical dislocation, percussion stunning, or lethal injection are typically used for smaller flocks, whereas containerized gas units and whole gas housing are often employed for larger flocks (34, 37). APHA will also perform the primary cleaning and disinfecting of the premises (36), and the premises will then be responsible for secondary cleaning and disinfecting at their own cost, which is approved as satisfactory by APHA. Twenty-one days after the completion of secondary cleaning and disinfection, new birds may be introduced onto the premises. Provided that the premises remains disease-free, once the timelines for the zones have passed, the zones can be removed and the area designated as free from disease.

#### 3.1.2 United States outbreak response processes

In the USA, each state has their own response plan to follow in the case of an outbreak. The federal government oversees this process and will participate in the outbreak response if invited by the state. When a grower suspects HPAI in their flock, they contact their veterinarian, who then contacts the state animal health official (SAHO) and the area veterinarian in charge (AVIC). Contact is then initiated with USDA Animal and Plant Health Inspection Services (APHIS) Veterinary Services’ district office (DO) for avian health. Following this, the AVIC and state animal health official (SAHO) will assign a foreign animal disease diagnostician (FADD) to attend the premises (35). This is a veterinarian that has completed specific diagnostician training with the National Veterinary Services Laboratories (NVSL) relating to foreign animal diseases (38). The FADD will contact the premises within 8 h, and schedule a site visit for within 24 h of assignment.

The FADD will perform physical examinations of the suspected infected birds, conduct sampling while on the premises, and submit these samples to the National Animal Health Laboratory Network (NAHLN), as well as recommend quarantine or restrictions to the SAHO and AVIC. The NAHLN is comprised of 60 state-level laboratories across the country that test for animal disease (39). While samples from suspected infected premises are sent to NAHLN, the NVSL Reference lab in Iowa must confirm HPAI before an outbreak can be declared. If the samples are positive for HPAI, the NVSL Director will notify Veterinary Services and the AVIC for the state, who will let the premises know that the results were positive (35). The state Department of Agriculture and United States Department of Agriculture (USDA) then publish the results on their websites, and the state and federal Public Affairs Agencies would collaborate to put out a press release confirming the outbreak. In addition, once an outbreak is declared, the federal government is responsible for notifying WOAH and implementing trade restrictions.

When a premises is determined to be positive for HPAI, several restrictions are put in place: an infected zone, which has a 3-km radius surrounding the infected premises, and a buffer zone, which has a radius of 7 km outside of the infected zone. The infected and buffer zones make up the control area. A surveillance zone is additionally implemented, which has a width of at least 10 km (40). Within the control area, restrictions are placed on the movement of poultry, poultry products, and potential fomites, and increased biosecurity measures are put in place (40).

Once a premises is confirmed positive for HPAI, the CVO of the USA will approve depopulation of the premises within 24 to 48 h of HPAI confirmation to prevent further spread. There are numerous methods to depopulate a flock, such as water-based foam, whole-house gassing, containerized gas, and cervical dislocation (41), although participants indicated that water-based foam and whole-house gassing were the most commonly-used methods, as these are the methods most applicable to larger barns. Unlike in the UK, where APHA conducts the primary cleaning and disinfection, cleaning and disinfection in the USA are typically conducted by the premises themselves or their hired subcontractors with supervision by the Cleaning and Disinfection Group, which is part of the federal disease response team (42). Premises must wait 14 days until they can apply to APHIS and State officials to restock with new birds; they must also undergo a final inspection and environmental sampling after the 14-day fallow period has passed. If they utilized outdoor composting as their disposal method, then this timeline increases to 28 days (43).

Table 3 provides an overview of the detection and response processes in place in the UK and USA. Both the UK and USA have a sequential multistep bureaucratic pathway, from initial suspicion to flock depopulation and clean-up. However, the larger size and greater priority of state processes in the USA adds an additional layer of complexity to their system.

**Table 3:** Summary and comparison of the response to suspected HPAI in the UK and USA.

Summary of HPAI response processes in United Kingdom and United States		
Step in the process	United Kingdom	United States
Report disease	Passive surveillance by poultry owners and veterinarians. Passive surveillance by park wardens and individuals upon finding dead wild birds.	Passive surveillance by poultry owners and veterinarians. Active wild bird surveillance by National Wildlife Disease Program.
Testing	Veterinary inspector checks birds for clinical signs and to collect samples. Samples are sent to Weybridge Laboratory.	FADD attends premises and collects samples. USDA APHIS Wildlife Services tests wild bird samples.
Confirmation	Weybridge Laboratory will confirm whether HPAI is present. CVO of affected country declares outbreak. UK CVO notifies WOAH.	NAHLN will test samples. Only the NVSL Reference Laboratory can confirm a HPAI outbreak. State Department of Agriculture and USDA publish results. USDA notifies WOAH.
Restrictions	Protection zone of 3 km radius. Surveillance zone of 10 km radius. Restriction zone with radius determined by risk assessment in cases of HPAI H5N1.	Infected zone of 3 km radius. Buffer zone with 7 km radius. Surveillance zone of at least 10 km.
Depopulation	Containerized gas units and whole gas housing employed for larger flocks. Cervical dislocation, percussive stunning, or lethal injection for smaller flocks.	Water-based foam and whole-gas housing employed for larger flocks. Containerized gas and cervical dislocation employed for smaller flocks.
Cleaning and Disinfection	APHA performs primary cleaning and disinfection. Premises performs secondary cleaning and disinfection at their own cost. APHA signs off on secondary cleaning and disinfection.	Cleaning and Disinfection group perform or supervise premises in cleaning and disinfection.
Disease Freedom	Can introduce new birds 21 days after completion of secondary cleaning and disinfection.	Can apply to introduce new birds 14 days after final inspection and environmental sampling carried out on premises. Must wait 28 days to apply to introduce new birds if outdoor composting was used as disposal method for carcasses.

### 3.2 Assessment of the outbreak response

Interview participants identified the steps involved in the response to HPAI and commented on how well this process is carried out. The availability of the equipment needed for depopulation, carcass disposal and disinfection, as well as the location of the infected premises, can affect the length of time it takes to carry out response activities.

Participants in both the UK and USA reported time delays due to the remoteness of locations of infected premises and the logistics of transporting the required equipment to those premises. In the UK, the Scottish Isles were viewed as particularly difficult to access for delivery of resources:

*“In Scotland, there is the geographical challenge that the majority of commercial poultry tends to be reasonably concentrated and in relatively well populated and accessible areas. But there’s poultry all over including the islands and highlands and from time to time, there’s challenging locations. When this happens, the quick timelines are stretched because there’s no presence in the islands.” – Participant 9, UK.*

In the USA, the vast distances needed to drive within states may affect the time it takes for operators to move the needed equipment for depopulation to the infected premises. The climate in each state may also influence the ability to implement certain depopulation methods. For example, the use of foam as a depopulation method requires a large water source (44). This is difficult to get access to in states where water sources limited or where the extreme heat or cold prevents the response team from being able to use them:

*“Foaming is one of the ways we depopulate… but if it’s minus 20, that foaming machine might not work. We now have generators and we have heating pads to make sure that the machine works, because we did not account for that when we started using this technology and you need a lot of water to produce this foam. This is going to freeze and damage the equipment.” – Participant 2, USA.*

Further, winter conditions can make it hazardous to drive on roads, especially far distances, so the safety of the response team may be prioritized over depopulation of an infected premises, leading to delays in culling a flock:

*“If you have a premises that come up positive in states like Wisconsin, North Dakota, South Dakota, or Minnesota in the depth of winter, those are very difficult responses. Remember, we are sending people, and they drive. They go out there, they risk their life, you know?” – Participant 2, USA.*

In the USA, participants indicated that some animal welfare organizations had voiced disagreement with different depopulation methods. For example, use of ventilation shut down was considered inhumane (45). The USDA only permits ventilation shut down as a depopulation method where other methods cannot be used or would not allow for flock depopulation within 24 to 48 h of being declared positive for HPAI (46).

Additionally, USA participants discussed the potential for bird owners to conduct the depopulation, disposal, and disinfection activities themselves. This does currently occur, and participants felt that this would have multiple benefits: the funds that the government would allocate to contractors to carry out depopulation would go toward the growers who had just lost their flock; depopulation could be done in a timely manner due to the individuals already being on the premises; and, this would mean less downtime for their business. However, this would require training for the premises and significant trust between the government and producers to ensure that all activities are being carried out thoroughly to not unintentionally spread disease.

In both the UK and USA, participants acknowledged that HPAI outbreaks take a large financial, emotional and mental toll on bird owners. Participants in the USA specifically were grateful for the indemnity offered to farmers when their birds were culled, but felt that the USA should continue supporting their farmers with financial and mental health resources when they experience an outbreak on their premises.

### 3.3 Stakeholder network map

**Figure 2:**
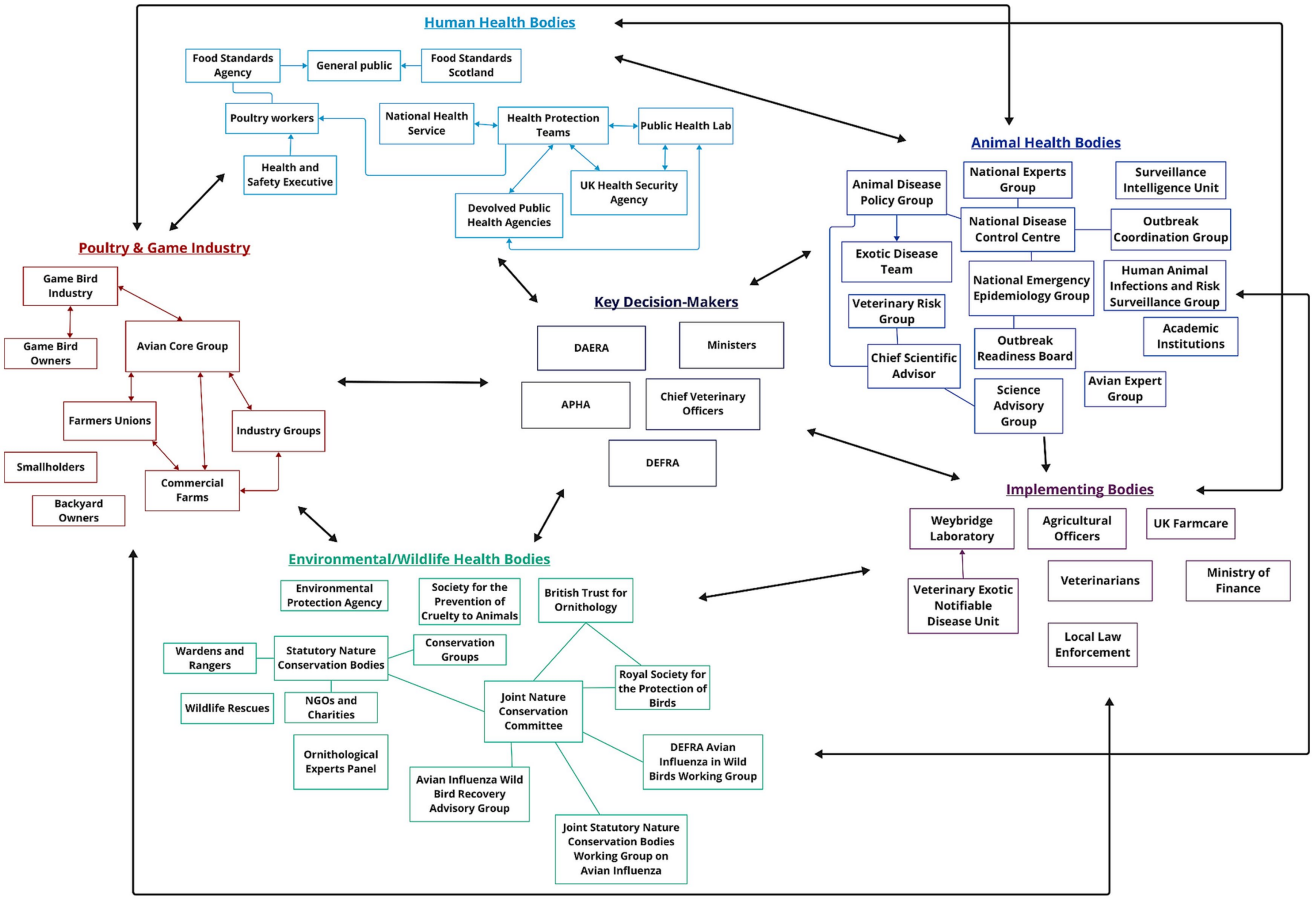
A map detailing the stakeholders involved in HPAI preparedness, decision-making and response activities in the United Kingdom. Stakeholders have been grouped according to their sector. Within each group, arrows indicate that information flows directly from one stakeholder to another, while lines indicate collaboration between the two.

**Figure 3:**
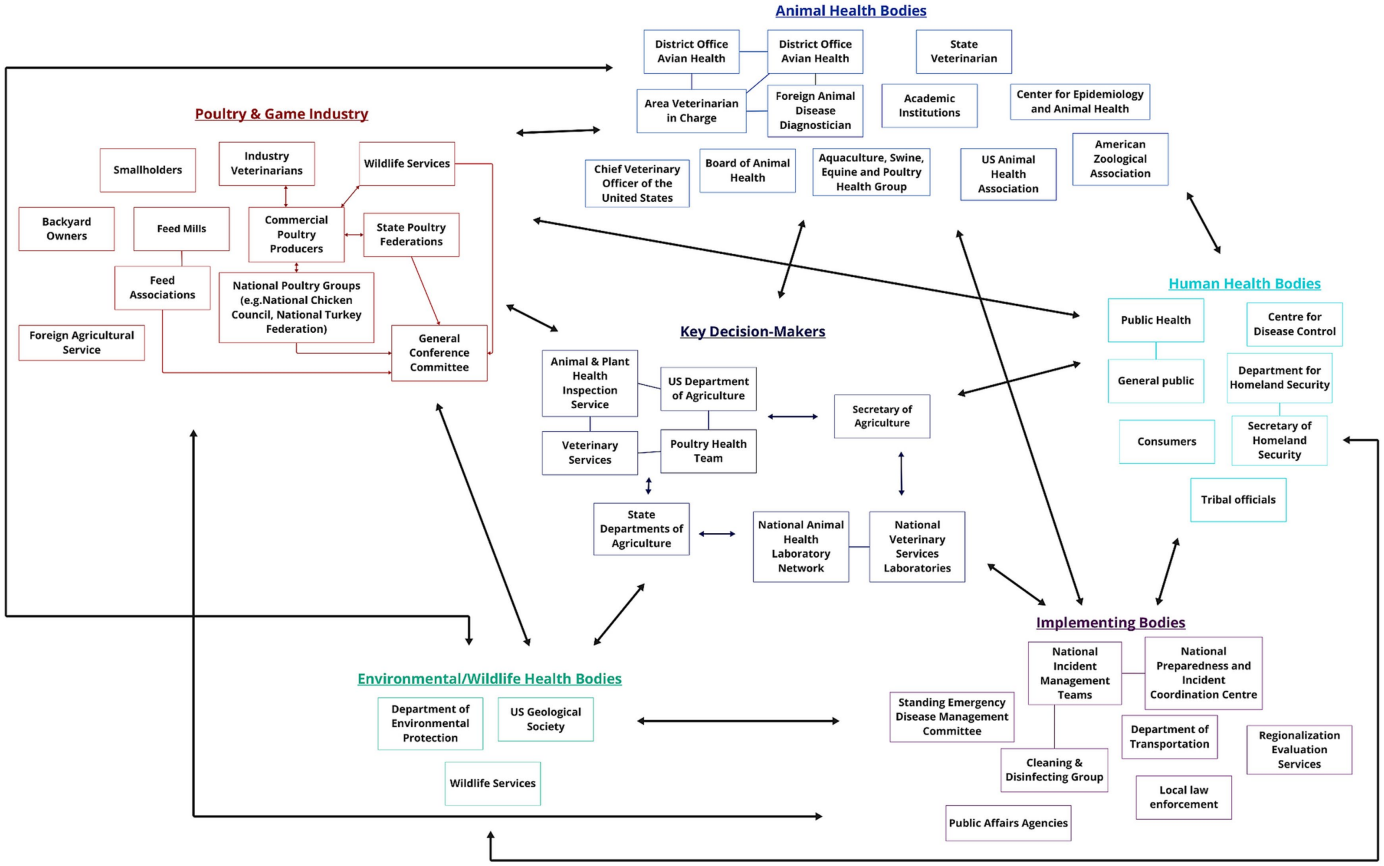
A map detailing the stakeholders involved in HPAI preparedness, decision-making and response activities in the United States. Stakeholders have been grouped according to their sector. Within each group, arrows indicate that information flows directly from one stakeholder to another, while lines indicate collaboration between the two.

Effective decision-making requires multi-disciplinary collaboration and expertise (47). Risk analysis framework requires involvement from diverse groups of stakeholders. It is therefore important to understand who is and is not already included at that science-policy-industry interface, how communication occurs between these stakeholders, and where there may be gaps or opportunities for better communication pathways. Stakeholder network maps based on participant information (Figures 2, 3) illustrate the stakeholder landscapes in the UK and USA.

#### 3.3.1 United Kingdom stakeholders

In the UK, participants identified APHA, Defra, as well as the CVOs of the devolved nations and Weybridge Lab as the most important and influential in HPAI outbreak decision-making. These stakeholders are responsible for the intake and feedback of communications, information, and data at the science-policy-industry interface. Information is typically shared with these decision-makers via risk assessment. Decision-making occurs through conference calls and working group meetings, and these decisions are then shared through meetings with stakeholder groups and online publications.

Numerous science-policy groups, such as the Animal Disease Policy Group (ADPG), National Experts Group (NEG), and the National Emergency Epidemiology Group (NEEG), exist within the animal health sector and act to provide science-based evidence for policy decision-making. The Minister of State for Agriculture and Food is responsible for signing off on any legislation or policies to be enacted.

Each of the science-policy groups within the UK animal health sector serve specific purposes. Once an outbreak is confirmed by the CVO, the National Disease Control Center (NDCC) is formed to manage the tactical outbreak response. This group is responsible for developing and interpreting policy within the established national framework, and preparing documents for Defra Ministers (48). Within the NDCC, the Outbreak Coordination Group (OCG), Animal Disease Policy Group (ADPG), National Experts Group (NEG), and NEEG are established (48, 49):

- OCG: comprised of staff members from the Contingency Planning Division within APHA. The OCG creates situation and summary reports for those implementing risk mitigation strategies, and makes sure that strategy and policy are actionable (48).
- ADPG: acts as a forum to discuss animal disease policy and issues. The ADPG, and prepares, presents, and reviews disease control policies, and advises Ministers on policy recommendations; this group often makes final policy decisions (50). The ADPG also ensures consistency across the devolved administrations with regards to animal disease policy.
- NEG: provides expertise and advice on HPAI for disease control policies to the devolved administrations. The NEG will additionally be asked to perform a logical examination of a case of HPAI if further wild bird information is needed for a policy decision (48).
- NEEG: conducts epidemiological investigations and contact tracing on infected premises. The NEEG provides epidemiological data to the CVOs of the devolved administrations, Defra, and supporting policy teams to assist with policy decision-making. The NEEG utilizes the epidemiological information gathered on farm during the initial visit to the premises to answer specific risk questions. The NEEG additionally sits on case conferences with the CVO (51).

Further advisory groups that exist within the government and assist with outbreak preparedness and response include the Defra Exotic Disease Policy Response Team, Outbreak Readiness Board (ORB), Veterinary Risk Group (VRG), Human Animal Infections and Risk Surveillance Group (HAIRS), and the Avian Expert Group (AEG). The Exotic Disease Policy Response Team was viewed by stakeholders as an essential stakeholder for managing, controlling and eradicating HPAI. This team, headed by Defra, makes policy decisions based on information provided to the CVOs and the ADPG. ORB is comprised of representatives from APHA, Defra, the NEEG, and devolved governments, and oversees HPAI preparedness and response activities. The VRG reports to the CVOs regarding the risk of potential threats to animal health and welfare, and provides advice that may help inform policy. This group prepares and reviews risk assessments to share with the devolved nations (52). The HAIRS is a multidisciplinary group that meets on a monthly basis in order to assess the risk of emerging zoonotic threats, such as HPAI, and prepares risk assessments which are then posted on the GOV.UK website (53). The AEG provides a forum for policymakers, epidemiologists, academic and scientific institutes, and APHA veterinarians to provide updates on HPAI outbreaks and offer expertise to Defra and APHA to assist with surveillance and decision-making (54).

The Ministry of Finance is a separate governmental department that plays a role in allocating funds for HPAI outbreaks and response activities. UK participants described the process of the devolved administration submitting a request for funds to the business support team, who would then formally request money from the Ministry of Finance in the form of a letter. These funds are then re-allocated from wherever there is a surplus within the government to support HPAI outbreak response activities.

There are additional groups that exist independently from the government but still assist with decision-making. The Science Advisory Council provides Defra with independent advice on science, policy and strategy for decision-making purposes (55). The Chief Science Advisor, Defra, and UK CVO also commissioned the Scientific Advisory Group in HPAI, which is made up of numerous experts in veterinary science, epidemiology, ecology, virology, ornithology, and social sciences. This group was charged with preparing an independent report assessing the current ongoing HPAI outbreak (56).

Participants viewed universities and academic institutions as playing an important role in surveillance, modeling, and risk mitigation. Research groups within these institutions have capability and expertise to study the virus and its transmission, model its spread, and determine potential methods to better minimize the risk of HPAI incursion. Academic institutions then share and disseminate this research either directly with members of government, or indirectly through peer-reviewed publications, and at conferences. In addition, participants described the role that veterinarians play in assisting poultry keepers with surveillance and biosecurity. Veterinarians are often the first to conduct a preliminary check on a premises when there is suspected HPAI, and decide whether APHA needs to be contacted. Veterinarians may also be the first to notice symptoms of HPAI during a visit to the holding for other reasons. Participants mentioned that veterinarians may work with both commercial and smaller premises to create a biosecurity plan, and are often responsible for assisting poultry keepers with surveillance and implementation practices. Veterinarians can therefore play a key role in detecting HPAI and preventing HPAI incursion on a premises.

Within the UK, detection of HPAI in wild birds is primarily dependent on passive surveillance, which relies on members of the general public and park wardens to report any suspicious cases. When Defra is notified of potential HPAI in dead wildfowl through their dedicated helpline for those who find dead wildfowl, APHA contracts UK Farmcare to collect wild bird carcasses and deliver them to APHA veterinary laboratories for testing, although this is the extent of the UK Farmcare’s role in an outbreak (49).

Natural England, Natural Resources Wales, NatureScot, and the Northern Ireland Environment Agency make up the Statutory Nature Conservation Bodies (SNCBs) in the UK, and were perceived by participants as the most important decision-makers at the environmental health interface. These three organizations are in constant contact with wildlife organizations, charities and non-governmental organizations to monitor the ongoing wild bird situation and provide biosecurity advice for areas with large populations of wild birds. These three organizations also provide weekly ornithological and wildlife disease expert advice to Defra regarding the ongoing HPAI situation (57).

The Joint Nature Conservation Committee (JNCC) is a conservation body that works in partnership with the devolved nations and working groups within the UK to provide national bird population monitoring schemes. The JNCC was commissioned to create an Avian Influenza Wild Bird Recovery Advisory Group for England and Wales, and additionally sits on the Defra Avian influenza in Wild Birds Working Group and the Joint Statutory Nature Conservation Bodies Working Group on Avian Influenza. The Avian Influenza Wild Bird Recovery Advisory Group was commissioned for creation by Defra and the Welsh Government, and is made up of experts from various bird conservation and wildlife organizations. This advisory group works with NatureScot to gather information to determine what conservation and monitoring actions need to be taken in response to HPAI in wild bird populations; this information exchange occurs during workshops, of which the resulting discussions are then published as a report. The Avian Influenza in Wild Birds Working Group brings together expertise from Defra, APHA, Natural England, and the JNCC. This group assists the devolved nations in collaborating in their approaches for mitigating the risk of HPAI in wild bird populations. This group then escalates discussed issues to the ADPG and Defra Ministers for decision. The Joint Statutory Nature Conservation Bodies Working Group on Avian Influenza is a group coordinated by the JNCC and is comprised of the Chief Scientific Officers and Directors from the SNBCs. This group allows for collaboration across the devolved nations with regards to implementing conservation bodies’ response activities for HPAI (58).

The Ornithological Expert Panel (OEP) is an advisory group chaired by APHA and comprised of ornithological and wildlife experts, as well as natural resource organizations, and provides veterinary and scientific information to APHA in response to policy questions (58). The Environmental Protection Agency (EPA) is additionally called on to provide support to APHA in the event of an outbreak. Participants discussed the Scottish EPA’s role in providing GIS maps to the Scottish Government with details of an infected premises and the surrounding areas. The Scottish EPA also provides APHA with advice about how to manage HPAI outbreaks while minimizing environmental impacts, such as by ensuring the proper disposal of contaminated manure, bedding and litter (59).

Other wildlife and environmental organizations play a role in monitoring the ongoing HPAI situation in wild birds and providing advice to the general public, such as the National Trust, JNCC, and British Trust for Ornithology (BTO) (57). The Royal Society for the Prevention of Cruelty to Animals (RSPCA) and the Royal Society for the Protection of Birds (RSPB) were described by participants as playing a small role in decision-making. The RSPB and other wildlife organizations monitor wild birds, patrol for carcasses, and report wild birds to APHA as part of the UK’s passive wild bird surveillance. The RSPCA and wildlife rescue services often advise the public on what to do when they come in contact with a sick or dead bird. As part of various wild bird population schemes, the BTO, RSPB and JNCC monitor different bird populations throughout the UK to provide a better understanding of the impact of HPAI on wild birds (60). The resulting census data and trends are published on JNCC, BTO, and GOV.UK websites (58). Participants felt that environmental groups would have the ability to provide advice to policymakers if a large wild bird population were to die due to HPAI. Many of these environmental and wildlife stakeholders are included in national training exercises to prepare for future HPAI outbreaks.

Due to the ongoing risk that HPAI could infect humans, stakeholders from the human health sector are included in communication with APHA, Defra and the CVOs. The UK Health Security Agency (UKHSA) works in consultation with the animal health agencies, such as Defra, APHA, and the NDCC, as well as the devolved public health agencies (UKHSA in England, Public Health Scotland, and Public Health Wales), in order to monitor the risk of HPAI to the general public. During an outbreak, the UKHSA will provide health advice and messaging to Defra and APHA. At the ground level, the local Health Protection Teams (HPTs) will lead the public health response in collaboration with Defra, APHA, local National Health Service (NHS) agencies, local authorities, and the devolved public health organizations. Defra will feed strategic information from the NDCC to the HPTs. Public health messaging regarding HPAI is shared on the UKHSA and devolved government websites for the general public to access (61). Similarly, the UK Health and Safety Executive (HSE) publishes guidance for reducing occupational exposure for those working with poultry or with the virus itself. HSE also provides guidelines for actions that should be taken in response to exposure to HPAI (62).

The Food Standards Agency (FSA) and Food Standards Scotland (FSS) are responsible for providing advice to the public on the risk of contracting HPAI through the consumption of poultry products (63). The FSA and FSS additionally sit on the ADPG and offers assistance and advice to Ministers regarding policy questions (50), and provides the Scottish Government with information related to the detection of HPAI in slaughterhouses. The FSA and/or the FSS will carry out risk assessments on request by the UK or devolved governments to determine risk to food safety, and these risk assessments then help inform legislative decisions and guidance for consumers and the general public (63, 64).

At the industry-policy interface, the Avian Core Group (ACG) acts as the key stakeholder that links the poultry and game bird industry with government decision-makers. Farmers unions, game bird industry representatives, and poultry industry representatives, such as the British Egg Industry Council (BEIC) and British Poultry Council (BPC), are appointed by APHA to sit on this group. Defra meets regularly with the ACG to provide situational and policy updates for these groups to share with their members, and the ACG is provides feedback to Defra on behalf of their members regarding policy decisions and implementation practices. Participants described the ACG as having the most influence on outbreak decision-making of any of the industry stakeholders. Poultry industry representatives additionally have the ability to contact Defra or APHA directly with specific questions or issues, but would normally communicate through the ACG meetings. Since backyard owners and smallholders are less likely to be represented by a farmers union or industry representative, they may not be afforded the same opportunities to receive updates from and provide feedback to the ACG as larger, commercial holdings. As such, backyard owners and smallholders may need to rely on other sources of information regarding HPAI outbreaks, its risks, and what can be done to mitigate those risks, such as word-of-mouth, government websites, radio or television advertisements, or social media.

Figure 2 provides an overview of the stakeholders involved in HPAI decision-making and response in the UK.

#### 3.3.2 United States stakeholders

Participants identified the USDA APHIS, as well as the states Departments of Agriculture, and National Veterinary Services Laboratories (NVSL) as the most important decision-makers. According to participants, the impacted state’s Department of Agriculture is responsible for carrying out the response to an outbreak; if they ask APHIS to participate in the response, then APHIS will oversee and finance the activities. In special circumstances, when the President of the USA formally declares HPAI an emergency, or if the Secretary of Agriculture requests assistance, the Department and Secretary of Homeland Security would take over the coordination of federal agencies and support while the USDA maintains leadership over the overall management of the outbreak (65).

Within APHIS, Veterinary Services (VS) is the primary department responsible for dealing with a HPAI outbreak. Their Poultry Health Team, which is a subset of the Aquaculture Swine Equine and Poultry Health group that is made up of veterinarians and program analysts, works on decision-making by developing the policies for responding to outbreaks of HPAI. National Preparedness and Incident Coordination (NPIC) is located within the Strategy and Policy side of VS, and is made up of experts in animal health emergency management. NPIC is responsible for providing the national guidelines for foreign animal disease outbreaks, referred to as the Foreign Animal Disease Preparedness and Response Plan. They additionally provide preparedness training through VS for HPAI outbreaks (66). NPIC oversees the creation of National Incident Management Teams (NIMTs), which provide the outbreak response. Participants described these teams as being set up in different areas across the USA and called upon to enact the outbreak response activities when there is an outbreak in their jurisdiction. If no teams are available in that given area, then a NIMT from another area may be recruited. The State Department of Agriculture would ask the NIMT to be deployed to assist with an outbreak if their resources are overloaded. The Cleaning and Disinfection Group that oversees the cleaning and disinfection of a depopulated premises is part of the Operations arm of the NIMT. NIMT is also responsible for other aspects of the outbreak response, such as epidemiological investigations of infected premises, depopulation, and carcass disposal (65).

There are several other departments within the USDA that play a role in HPAI outbreak preparedness and response, including the Center for Epidemiology and Animal Health (CEAH), Foreign Agricultural Service (FAS), and Regionalization Evaluation Services (RES). CEAH is a science center located within VS, and is directly involved with HPAI decision-making (67). Participants defined the CEAH as responsible for delivering animal health analyses and risk assessments to government and industry decision-makers so that these parties can then make informed decisions. FAS, in contrast, works on the international side by assisting foreign officials on their HPAI outbreak response and sharing scientific information (65). RES additionally works on the international side of analysing animal health in foreign zones and compartments, and determines the risk of disease incursion due to importing from those regions (68). Additionally, in 2024, the Food Safety and Inspection Service (FSIS) began testing dairy cattle carcasses for H5N1 influenza A virus in response to the outbreak of HPAI in dairy cattle across numerous states, adding this testing to their existing surveillance program within slaughterhouses (69).

At the state level, respondents provided insight into numerous organizations that work to ensure timely and effective decision-making and response. The Departments of Agriculture at state-level are responsible for overseeing the entire outbreak response if it occurs within that state. In some states, such as Minnesota, the state Board of Animal Health takes on this role instead. Each state is required to have a plan for responding and containing disease outbreaks that is approved by APHIS. This plan includes the creation of a Standing Emergency Disease Management Committee, who host meetings and disease training exercises and coordinate with any impacted tribal governments [National (70)].

The Departments of Transportation play a role in the response when trucks transporting heavy equipment or carcasses are involved. They provide maps with weight load limits to the state Department of Agriculture. Each state’s Department of Environmental Protection or Quality provides information to the State Department of Agriculture about the numbers, and methods by which birds are depopulated and disposed, thereby giving them the opportunity to impact decision-making and outbreak response strategies.

In the USA, active wild bird surveillance is conducted as part of the National Wildlife Disease Program. Samples are collected and tested for HPAI by the USDA APHIS Wildlife Services, who collaborate with universities and other academic institutions to conduct surveillance; the results are posted online to illustrate areas where there may be increased risk of HPAI incursion (71). Participants also indicated that the US Geological Services conducts wild bird testing. The American Zoological Association similarly coordinates with APHIS to implement active and passive surveillance for their properties and exhibits (65).

At the local level, county, state or local law enforcement would be enlisted by the Poultry Health Team of APHIS if people on the infected premises were not complying with or were threatening the outbreak responders. While participants said that this did not happen for every premises visited, law enforcement were needed more often than responders would like. In addition, tribal governments have jurisdiction over their own areas, which includes enforcing their own laws; however, tribal governments will work in collaboration with state and federal officials, as well as universities and academic institutions, to manage agricultural issues, including disease outbreaks (65).

Due to the potential zoonotic risk of HPAI, at the time of interviews, the U.S Centers for Disease Control (CDC) was providing weekly updates on their website regarding the number of HPAI cases in poultry and the risk to humans. These updates along with human cases are currently provided by the American Medical Association. The CDC, APHIS, and local public health departments work closely to monitor response workers for flu-like symptoms (65). The CDC has collaborated with the USDA to prepare HPAI-related materials for a range of audiences, including the general public, poultry workers, and healthcare workers (72). Despite this, participants did not feel that the CDC played an active role in HPAI response, but rather that the CDC was seeking situational awareness in case of zoonotic infection.

The National Poultry Improvement Plan (NPIP) is a voluntary scheme that links the federal and state governments with the poultry and game industry to tackle disease threats [National Poultry Improvement (73)]. APHIS heads the NPIP, and this program provides routine disease surveillance and standards for poultry premises to follow, such as for biosecurity. As part of the NPIP surveillance, poultry producers conduct sampling of their flocks and submit these to NPIP-approved laboratories. This program also allows for communication between the poultry industry, state veterinarians, and APHIS through the General Conference Committee, a committee that advises the Secretary of Agriculture on how best to assist the poultry industry with issues pertaining to poultry health and disease. A conference is held every 2 years where the poultry industry can specifically provide feedback on policy decisions.

One of the primary reasons for industry to join the NPIP is because in the event of disease and depopulation, producers receive 100% indemnity for their culled flock. However, since the NPIP is voluntary and there is a price to joining, backyard farmers and smallholders tend to not enroll as there is no incentive for them if they only wish to sell their poultry products locally.

Within the poultry industry, other impacted stakeholders mentioned by participants included industry associations and feed mills. Industry associations include the National Chicken Council and National Turkey Federation at the federal level, and State Poultry Federation at the state level. These associations often have subject matter experts or employees that work specifically with USDA APHIS during an outbreak, and play a role in decision-making by providing feedback on outbreak policy decisions. The federal organizations will also lobby on behalf of their producers. With regards to feed mills, during an outbreak of HPAI, feed mills lose revenue due to the mass depopulation of poultry, as once this occurs, feed is no longer needed until the premises places new birds. Lastly, the US Animal Health Association (USAHA) works with poultry producers, federal and state governments, universities and research institutes, and veterinarians to provide solutions to animal disease outbreaks. The USAHA hosts a national yearly forum for members to meet, discuss current issues, share new information and ideas, and collaborate on approaches to controlling livestock diseases (74).

Figure 3 provides an overview of the stakeholders involved in HPAI decision-making and response in the USA.

### 3.4 Areas for better collaboration

Numerous stakeholders are involved in the HPAI outbreak response implementation and the decision-making that underlies this process. Participants from the UK and USA specifically provided examples of how these countries had sought to improve collaboration across different sectors. Many participants in the USA praised the creation of the NPIP, which is a voluntary collaborative program between the federal and state governments and poultry industry. This program provides a common ground for the government and industry to eradicate diseases, such as HPAI. However, some noted that while the NPIP provided a platform for the poultry industry to give feedback to policymakers, the process of enacting proposed changes took a long time, if their feedback resulted in change at all. This can make participants feel disincentivised to propose any changes, as by the time they are put in place, they may not be relevant anymore. An example of this timeline was given by one of the USA participants:

*“Let us say that I want to make a change in the NPIP, and let us say that we have got a biennial conference coming up this summer. I usually have to have my resolution prepared and submitted to the NPIP office the previous winter. Now there are provisions to submit proposals or resolutions on an emergency basis, but it has to be approved by essentially the executive committee of the National Poultry Improvement Plan to get on the agenda for the biennial meeting. So the NPIP decides, yes, we have passed this resolution, we want to do X. That proposal goes back to the USDA, the USDA has to pass it around to all the individual entities within the USDA, and then they have to publish it as a rule. Now to get all those reviews done and to actually get the thing printed and out there has taken anywhere from an additional eighteen months to two years.” – Participant 3, USA.*

Participants from the UK and USA expressed a need to better include smallholders and backyard owners in communications and outbreak planning. In the UK, the Avian Core Group exists as a government-led group responsible for sharing HPAI-related information with poultry bodies and organizations and accepting feedback from these groups. Smallholders and backyard owners tend to not be represented on the Avian Core Group and miss out on opportunities to receive updates and provide feedback on their experiences. USA participants similarly discussed the exclusion of backyard owners and smallholders from the existing communication networks at the policy-industry interface, as it would be difficult to reach out to every smallholder and backyard owner. There is also the view that these poultry owners are less educated on HPAI than the commercial sector, and so would require more outreach to educate them, which would be a timely endeavor.

Distrust between different stakeholders was identified as a common theme in the UK and USA. Participants from the UK discussed the distrust that can occur between government and smallholders / backyard owners. Smallholders and backyard owners can feel that individuals at the government level do not have an understanding of ground level activities and poultry industry perspectives, while government feels that bird owners are not implementing strict biosecurity measures and do not appreciate the economic impacts of their actions on commercial farms. Follow-up interviews in the UK indicated that Defra had worked to bridge this relationship, but maintaining effective communication and collaboration at the policy-industry level remains important.

A couple of participants in the USA expressed a distrust in the CDC following the COVID-19 outbreak response. There were concerns that if HPAI were to become zoonotic and cause an outbreak in humans, the CDC would be unable to efficiently deal with this outbreak:

*“CDC did a very nice job of bungling the response month after month involving human disease. I cannot imagine the mistakes that they would be likely to make involving a disease that moved back and forth between animals and people.” – Participant 3, USA.*

USA participants also described scenarios where a US state may not trust another state to have carried out all HPAI-related procedures and as a result, block imports across state lines. However, this was mentioned as rarely occurring as states tended to cooperate well with each other and follow outbreak guidelines. Most participants expressed pride in how well their federal, state, and industry stakeholders regularly collaborated.

### 3.5 Improving information sharing

Decision-making and implementing response activities during an outbreak of HPAI requires data and information sharing between stakeholders from various sectors. One issue that UK participants working at the science-policy interface brought up was the delay in data sharing occasionally experienced by scientists when asked to undertake a project for the government. While there are often data sharing agreements put in place to help make data sharing conducive, there can be lengthy delays between determining that this agreement needs to be made and it being implemented by government, especially if multiple governmental bodies need to approve this agreement. During an outbreak, this issue may be further compounded due to policymakers being busy with the outbreak response, leading to a delay in sharing data and therefore in scientists being able to provide risk assessments in return to policymakers. Participants felt that it may be government regulations and security protocols causing this delay in data sharing with scientists, which in turn impacts scientists’ ability to complete requests in a timely manner:

*“Sharing data with researchers is more difficult and regulated, so cannot be done rapidly.” – Participant 4, UK.*

The National Poultry Register is a UK government database where individuals with 50 or more domestic birds on their property are required to register their flocks. While this register went unmentioned by most participants, two UK participants felt that there were several ongoing issues with this approach. There was concern that the data on the National Poultry Register was not current, as individuals register their birds when they first receive their flock but may not update the register as mortalities or births occur. In addition, the registration requirement of the National Poultry Register only applies to those with 50 or more birds. This means that backyard owners and smallholders may not voluntarily register, so the register is likely missing data. This makes it more difficult to obtain information on the location of small flocks, types and numbers of birds, all of which are important datasets to have during HPAI prevention and outbreak response. Having a full understanding of the poultry populations across the UK allows for easier contact tracing, virus mapping, and the ability to warn all poultry owners when HPAI is present in their area:

*“People remember to register when they have poultry, but forget to take themselves off the register when they no longer have poultry. Keeping the register up to date and valid is important for APHA and investigations, but it is not always up to date.” – Participant 11, UK.*

USA participants suggested that the information available on government websites regarding HPAI tended to cater to larger commercial holdings as opposed to smallholders or backyard owners. The information currently shared on government sources may not be accessible or usable for non-commercial farmers, or contain information regarding biosecurity that is not relevant to smaller holdings.

Some UK participants described the communication pathways between government decision-makers and poultry workers and producers as indirect. This is due to the numerous steps and personnel involved in passing down communications on a commercial premises, as information may need to pass through the ACG, poultry associations, commercial farm owners before it reaches the individuals working on the ground with poultry. The end result of these communication pathways is that by the time the messaging reaches these poultry workers, it may have changed or been watered down; an example of this includes the importance of biosecurity as the best preventor of disease incursion not being effectively transmitted once it reaches poultry workers responsible for following on-farm biosecurity practices. This indicates a need to minimize the number of steps in this communication pathway and ensure that poultry workers are not receiving second-hand information. One participant provided an example of how these steps in communication may lead to inaccurate messaging:

*“Information cascades down through the company directors. This is filtered down to an area manager; they are not always good at passing it on, so you get some slippage occurring. This then comes down to farm managers… Sometimes what’s communicated to producers is coming from secondary routes or people who are picking up the information themselves second-hand.” – Participant 8, UK.*

Participants in the USA described the ability of the state of certain states to maintain consistent messaging regarding HPAI across the different governmental departments and organizations websites. This is possible when the Board of Animal Health, veterinarians, and other state agencies ensure that messaging is consistent across all departments when queries arise. Participants felt that providing this consistent messaging meant that those seeking information would be less likely to be misinformed or experience confusion over differences in messaging. However, other USA participants identified the challenge of directing non-government individuals to government websites. While the HPAI-related information is available on government websites, many laypeople do not know where to find it which can lead to the risk that they receive their information from a non-reliable source:

*“We have a public website where we post our written policy once it’s been cleared, so anybody can see that, it’s just a matter of knowing where to look and then reading it.” – Participant 8, USA.*

This may demonstrate a need for more active communication approaches for smallholders and backyard owners or improve usability of the platforms on which this information is hosted. UK and USA decision-makers tend to post situational updates and share information via government websites, although UK participants mentioned that radio stations, newspapers, social media, and other types of media were being used or considered in order to reach smallholders and backyard owners in more remote locations. In addition, the USA and UK both actively share situational updates and decisions through the Avian Core Group in the UK and the NPIP in the USA. This suggests that both nations are already utilizing both active and passive communication strategies to reach the different members of the poultry industry; however, the active communication methods being employed in the UK to reach smaller bird owners may not be effective and should be evaluated to ensure they are reaching their target audience.

This participant comment may also suggest that there needs to be increased searchability or a better user experience of the platforms on which this information is hosted. This is corroborated by UK participants. Within the UK, GOV.UK has been utilized as a central platform to share information and updates on HPAI outbreaks in the country. UK participants appreciated that this provided consistent messaging but felt that the website overall was not user friendly. Participants cited the numerous different pages they had to search through to find the information they were seeking as particularly frustrating and suggested that information be consolidated onto fewer pages within the GOV.UK website to create a better user experience.

### 3.6 Outbreak expertise

Outbreak expertise refers to the knowledge and experience required for outbreak decision-making and response implementation. Participants in the UK described the recent inclusion of in-house ornithological expertise by government decision-makers as a positive step, as this was viewed as an important viewpoint to incorporate at the science-policy interface, particularly for preparedness work regarding wild birds.

In the UK, two challenges were identified within the theme of outbreak preparedness. The first was the movement of policy advisors between departments within the UK government. Expertise is typically gained through experience; however, movement within the government hinders the ability for somebody to remain in a position long enough to gain that necessary experience:

*“With each outbreak, everyone learns more, but as time passes, people retire or move around, so it’s important to have the expertise that can deal with each of the outbreaks.” – Participant 10, UK.*

The second challenge was a lack of HPAI-related poultry knowledge by general veterinarians. Participants mentioned that general veterinarians are often a source of information for smallholders and backyard owners, but due to their lack of experience with poultry and HPAI, they may not be able to provide reliable advice regarding HPAI and biosecurity:

*“Particularly for smaller units, support comes from a general vet, not from a specialist poultry practice. So there’s some vets that have a very good understanding of biosecurity and the practical implications and what’s being proposed, but some of the advice that’s been given is not workable, and as a consequence the advice may be given but not acted upon or understood.” – Participant 8, UK.*

Participants in the USA indicated a similar problem with retention and lack of expertise. Due to the high stress nature of outbreak response activities, employees working during an HPAI outbreak are more likely to experience burnout and leave their positions. This means that the experience gained by those personnel is lost and new employees must take on the outbreak without previous experience. In addition, due to the quick pace of actions taken during an outbreak causing time restraints, it is difficult to properly onboard and train new employees:

*“I think getting people on boarded and not burning out people would be very helpful.” – Participant 6, USA.*

### 3.7 Preventing future disease incursion

Strict biosecurity at the farm-level is viewed by participants in both the UK and USA as the most important risk mitigation strategy for poultry premises; failure to implement or follow biosecurity measures increases the chance of a HPAI incursion on the premises. Examples of biosecurity measures include restricting the number of visitors, designated protective clothing and footwear for entering a premises, and disinfecting any equipment before it enters or exits the premises (75). UK and USA participants expressed concerns that bird owners may not fully understand the risks of contracting HPAI, leading to relaxed on-farm biosecurity and further outbreaks. For example:

*“There may be a lack of understanding of housing measures. People feel there’s no outbreaks near them or they do not have contact with wild birds, so they can keep their birds outside. We need to educate people on risks.” – Participant 3, UK.*

This provides an example of the lack of understanding of how quickly HPAI can spread and why on-farm biosecurity practices, such as housing birds, may be relaxed due to perceived low risk. In addition, it was acknowledged that due to the number of personnel on a given premises, individual compliance with strict biosecurity may differ across and within premises, thereby increasing the risks of a HPAI outbreak and demonstrating a need for education at the individual level:

*“There’s a sense of ‘we did not get it last time or the year before, so it’s just not going to happen’.” –Participant 8, UK.*

Participants in the UK described numerous types of simulated practice outbreaks to prepare for future occurrences. APHA and the NEEG are two groups that run these contingency scenarios within the UK to determine where there are areas for improvement before an outbreak occurs. Outbreak exercises are also conducted in the USA by APHIS Veterinary Services to provide stakeholders involved in outbreak response, such as federal, state, and local responders, the poultry and game bird industry, and academic institutions the opportunity to practice the response activities that need to take place (65). These practice outbreaks were perceived positively by participants as effective measures for outbreak preparedness.

Participants from the USA expressed pride in their active wild bird surveillance program, which covers the four flyways that see the movement of migratory birds potentially carrying HPAI into the country. This surveillance program is viewed as extremely useful in predicting where the virus is likely to be located or spread, and was described by several participants as being the best in the world:

*“We’ve got wild bird surveillance at all four flyways. It is detecting the virus. It is giving us a really good picture of how much virus is circulating and which types of species, and it is publicly available.” – Participant 8, USA.*

However, some UK and USA participants expressed concern that having increased predictive knowledge of future HPAI outbreaks would not necessarily improve preparedness. They felt that strict biosecurity was the best method for reducing the risk of HPAI incursion and sharing wild bird surveillance data would cause bird owners to relax their biosecurity when they felt the risk of incursion was low. In addition, while determining risk factors for a HPAI outbreak on a premises is useful, UK and USA participants felt that many of the actions that could be taken to lower the risk were not feasible for bird owners. For example, a farm may need to add or update facilities, or change the layout of their barn or where the entrances are. Available funds are often needed to be able to carry out these preventative measures, and for many farmers, this is too costly to undertake:

“Farmers need to change the layouts of their farms, where their entrances are, new draining, washing facilities, where the vehicle washing facilities are; only top commercial places could afford all these changes.” – Participant 2, UK.

## 4 Discussion

The objective of this study was to determine how key stakeholders involved in HPAI prevention and response in the UK and USA perceived their country’s outbreak decision-making and response processes and how they could be improved in preparation for future outbreaks. Interview participants were initially recruited using known contacts of the primary investigators and then using a snowball approach to expand the participant list. While this allowed for ease of recruiting individuals whose work and experience related to HPAI preparedness and response, it also meant that recruitment was limited to participants’ networks.

This study examined the UK and USA, two developed countries with well-established commercial poultry industries, having produced 1.95 million and 22.03 million tonnes of poultry meat in 2022, respectively (76). The USA specifically is the largest producer and second largest exporter of poultry products globally (77). In contrast to the UK and USA, lower- and middle-income countries may rely less on commercial poultry industries and more on local and backyard poultry production for household income and nutrition (78). However, while these lower income countries may not share the same communication pathways between commercial poultry industry leaders and government bodies as the UK and USA, the recommendations for collaboration between those working on the ground with poultry and those deciding animal disease policy applies. Other members of WOAH will likely follow comparable procedures to the UK and USA when suspected HPAI is reported, but with their own set of rules around who specifically carries out each step. Previous studies have drawn similar conclusions regarding the need for open communication channels and discussion between government decision-makers and those within the poultry sector, and how this relationship can improve the likelihood of reporting suspected HPAI to the appropriate authorities (79, 80). Future studies could map the stakeholders and communication chains involved in less developed countries to determine the challenges specific to that country’s HPAI outbreak response systems.

UK and USA stakeholder maps were created using interview participants’ perceptions of who is involved in decision-making and implementation during a HPAI outbreak. Since countries may continually adapt and revise how they manage outbreaks to better mitigate the spread of HPAI, there was possibly significant variation across participants’ knowledge of the stakeholders involved in the process. Mapping stakeholders is a useful exercise to identify opportunities to improve communication and resilience across the system. Differences between the UK and USA stakeholder maps illustrate a greater number of key decision-makers identified in the USA, and a larger number of environmental/wildlife health bodies involved in UK HPAI decision-making and outbreak response. It should be noted that this study did not examine the specific differences between each US state, as this was beyond the scope of the project. Since the state is largely responsible for the overall disease response, each state may have its own variances in outbreak response. Future studies could broaden their sample to incorporate various state perspectives and participants from different sectors of the poultry industry.

In the UK, having a fewer number of key decision-makers suggests a more streamlined approach to overall decision-making in the UK, as there are fewer stakeholders to consult with when making final decisions. This may also suggest that these key stakeholders in the UK have a wider remit and scope of responsibilities. Having more decision-makers involved in the process who need to be consulted with could delay decision-making and response implementation. With the UK’s greater number of environmental/wildlife health groups, this could conversely indicate more red-tape and delays in decision-making and information due to the large number of stakeholders to communicate with. In addition, having a wide range of stakeholders within the environmental/wildlife health sector with different areas of expertise and competing priorities could lead to disagreements and inconsistency in messaging (81).

Having a larger number of decision-makers and working groups within a given sector could, however, also provide an avenue for better information sharing and outreach among these stakeholders, as a larger number of these stakeholders means they can encompass a wider audience within the environmental/wildlife sector. Boden et al. (47) emphasize the importance of multi-disciplinary stakeholder groups on effective risk governance, which suggests that having numerous working groups that include numerous stakeholders within the environmental/wildlife health sector could be beneficial for the UK’s management of HPAI outbreaks. Furthermore, due to the high intensity of the HPAI outbreak response as mentioned by numerous participants in both the UK and USA, having several key decision-makers in the USA with specific functions to spread the workload among may lessen the burden and prevent the response processes from getting overwhelmed and therefore delaying decisions that need to be made. Whether having increased stakeholder groups involved in decision-making and outbreak response implementation is positive or negative, it is important in either case that there is effective communication between all stakeholder groups and that challenges are mediated to ensure effectiveness of the country’s risk governance strategy (23).

Animal disease risk governance frameworks encourage cross-disciplinary collaborations between stakeholders (23, 82). Participants in the UK and USA described communication pathways between different governmental departments and science-policy stakeholders, but identified gaps at the policy-industry interface. Specifically, participants in both countries discussed a lack of information sharing with smallholders and backyard farmers, and a general distrust by policymakers in these premises to properly implement biosecurity at the farm-level. A study conducted with Scotland poultry owners with fewer than 50 birds found that premises tended to implement at least one biosecurity measure, but overall, their biosecurity was not comprehensive (83). These results are corroborated by Derksen et al. (84), who found that backyard owners in the USA tended to report contact between their flocks and wild birds and the poor application of biosecurity measures.

However, while concerns that farm-level biosecurity is not being properly implemented may not be entirely unfounded, noncompliance could be due to a lack of communication on behalf of the government. For example, in a study examining the perspectives of 18 backyard farmers in Canada, participants felt that the majority of backyard flock health issues were due to a lack of husbandry information, veterinary support, and slaughter facilities. In response to the 2004 HPAI outbreak in Canada, these participants described the stress they experienced due to not fully understanding the decision-making process with regards to testing, depopulation, compensation, and licensing. Furthermore, when asked for their perspectives on government, the key themes identified were distrust, lack of access to information, and government not respecting backyard farmers (85). This study was conducted in Canada, but the results could be extrapolated to explain why backyard owners and smallholders may not implement the same biosecurity measures that government feels they should.

Ensuring trust and transparency between government and bird owners is also imperative to HPAI reporting, as early detection of the virus relies on premises recognizing HPAI symptoms and reporting suspected cases to the appropriate authorities (86). A study conducted in the Netherlands by Elbers et al. (87) found that confusion around clinical signs of HPAI, lack of trust in government, and a lack of transparency in the notification procedures and reporting process were among the reasons that farmers would be inclined not to report suspected HPAI. This once again highlights the need to maintain relationships at the policy-industry interface, as rapid detection and response cannot take place if bird owners are not inclined to report HPAI in the first place.

The UK and USA both have initiatives aimed at sharing information with backyard farmers and small holders and encouraging the implementation of biosecurity. Defra hosts regular webinars titled ‘Stop the Spread’ for bird owners to learn how to best prevent HPAI on their premises and what biosecurity measures they should be undertaking (88). Similarly, ‘Defend the Flock’ is a program run by the USDA aimed at anybody who owns or works with birds. This program provides information regarding the importance of biosecurity and how to properly implement it (89). Given that interview participants indicated that both the UK and USA governments had made efforts to improve relationships with bird owners, follow-up research should be conducted to determine the strength of these programs and whether there has been improvement in farm-level biosecurity application by backyard owners and smallholders.

In an attempt to improve stakeholder communications, Defra has established an Avian Influenza Outbreak and Biosecurity Communications Stakeholder Group, which hosts organizations that represent backyard owners and hobby farmers, as well as commercial farmers, specialist bird keepers, and wild bird non-government organizations. The purpose of this group is to provide situational updates during HPAI outbreaks, as well as information regarding HPAI prevention and risk mitigation practices (58). While this group was formed in 2021, UK interview participants did not appear to be aware of this working group. This could indicate that further promotion needs to be conducted to increase awareness among the targeted audiences, or that the participants included in this study do not fall within the scope of the working group so would be unable to speak to the group’s purpose and impact.

While risk governance processes often rely upon policymakers and risk assessments, enacting these processes requires collaboration and input from a wide variety of impacted stakeholders across the science-policy-industry interface. This is supported by the new global strategy published by the Food and Agriculture Organization of the United Nations (FAO) and WOAH, who call for inclusion of all impacted sectors in HPAI control strategies, including the agricultural, public health, and environmental sectors (90). The UK and USA have established procedures to deal with a HPAI outbreak, but risk governance processes may not always function as intended. It is therefore important that the UK and USA ensure transparent communication and collaboration among stakeholders involved in HPAI outbreak processes so that all decision-makers and response implementers can carry out their role in preventing a HPAI incursion and maintaining disease-free status in their country. Continuous engagement and inclusion of all relevant sectors is a priority to ensure good risk governance processes in the prevention and management of HPAI and other zoonotic diseases.

## Data Availability

The datasets presented in this article are not readily available because of a possibility of de-anonymisation due to the participant sample size. Requests to access the datasets should be directed to Kimberly Lyons, klyons@ed.ac.uk.
