# Supplementary material for: Assessing stakeholder inclusion within high pathogenicity avian influenza risk governance strategies in the United Kingdom and United States
Source: Front Vet Sci. 2025 Apr 17;12:1547628. doi: 10.3389/fvets.2025.1547628 (PMC12043875; doi:10.3389/fvets.2025.1547628)
Supplement: Supplementary file 1 [file Table_1.docx]

**Supplementary Table 1.** Questionnaire developed and used for qualitative interviews.

| **Interview Guidelines.** | |
| --- | --- |
| 1. **Background** | |
| **Question** | **Notes** |
| A1.What is your current role or job description? |  |
| A2.What is your area of expertise or specialisation? |  |
| A3.How does your role relate to Avian Influenza outbreak preparedness and response in your country? |  |
| A4.Do you work alone, or as part of a team? Please describe |  |
| A5.Within which country do you work/live? |  |
| A6. Are you involved in AI outbreak preparedness and response plans of other countries? If so, which? |  |
| 1. **Identifying Stakeholders: Decision-making about AI outbreak preparedness and response** | |
| **Question** | **Notes** |
| B1. Which stakeholder(s) have the authority and responsibility for decision-making in response to an AI outbreak? |  |
| B2. Are government-based stakeholders associated with AI outbreak response primarily based within the same ministry/ department/ division and if so, which? |  |
| B3. Which other policy areas are typically involved in AI outbreak preparedness and response (beyond the relevant ministry/department/ division responsible for agriculture, food security and veterinary services)? |  |
| B4. Please describe the steps in the decision-making process (and stakeholders involved) with respect to a declaration of an AI outbreak and who has the final authority over the decision taken. |  |
| B5. Please describe which stakeholders are the **most** important and the **least** important in **decision-making** with respect to AI outbreak preparedness and response activities? Please comment on why you have prioritised stakeholders in this way. |  |
| B6. What data, information or intelligence (eg disease-related, resource-related, other services such as police etc, media etc.) are required for AI outbreak decision-making and who is responsible for knowledge creation and sharing? |  |
| B7.  Who is responsible for data/intelligence sharing and how is it shared? (e.g. formal/informal meetings, access to online secure platforms etc.) |  |
| B8. What other inputs are required for decision-making (e.g. expertise/capacity, financial etc.)? |  |
| 1. **Implementation of AI outbreak preparedness and response activities** | |
| **Question** | **Notes** |
| C1. Which stakeholder(s) are responsible for the implementation of AI outbreak preparedness and response policies and activities? Please consider institutions, organisations, individuals in government, industry, private sector, third sector, non-commercial farming communities, and general public  etc. |  |
| C2. Please describe which stakeholders are the **most** important and the **least** important in the **implementation** of AI outbreak preparedness and response activities? Please comment on why you have prioritised stakeholders in this way. |  |
| C3. What data, information or intelligence are required for the implementation of AI outbreak preparedness and response activities? |  |
| C4.  Who is responsible for data/intelligence sharing and how is it shared between stakeholders involved in implementation? (e.g. formal/informal meetings, access to online secure platforms etc.) |  |
| C5. How (or through what processes/structures) do these stakeholders receive and feedback information to decision-makers? |  |
| C5. What other inputs are required for decision-making (e.g. expertise/capacity, financial etc.) |  |
| C6. Are there any geographic-specific constraints on implementation (local, regional, national actors/regulations) etc. |  |
| C7. Are there key differences with respect to importance and influence of certain stakeholders within and outwith an outbreak? Please describe |  |
| C8. Are there any stakeholders or groups who are not currently involved in implementation, but may be impacted by AI outbreak preparedness and response activities? |  |
| C9. Are there any differences in implementation of preparedness and response activities in the event of high pathogenic vs low pathogenic outbreak? |  |
| 1. **Engagement with model and any further contacts** | |
| **Question** | **Notes** |
| D1. What types of information regarding wild bird incursion of AI do you think are most useful in informing AI outbreak preparedness and response? |  |
| D2. When considering the decision making and implementation process as a whole, what are the timelines typically between a decision being made and then it being implemented? Does this differ during or between outbreaks? |  |
| D3. How do you think vaccination fits into AI preparedness outbreak response? What is its’ current role and do you anticipate any changes in this role? |  |
| D4. What evidence would improve early warning and detection of AI, and how could this evidence be best communicated? |  |
| D5. Is there anyone else that you would be happy to put me in touch with to interview regarding AI outbreak preparedness and response? |  |

**Supplementary Table 2.** A list of abbreviations used throughout the manuscript.

| ACG  ADPG  AEG  AI  APHA  APHIS  AVIC  BEIC  BPC  BTO  CDC  CEAH  CVO  Defra  DO  EPA  EEAD  FADD  FAO  FAS  FSA  FSS  HAIRS  HPAI  HPT  LPAI  NAHLN  NDCC  NEG  NEEG  NHS  NIMT  NPIC  NPIP  NVSL  OCG  OEP  ORB  RES  RSPB  RSPCA  SAHO  SEPA  SNCB  UK  UKHSA  UKHSE  USA  USAHA  USDA  VENDU  VRG  VS  WHO  WOAH | Avian Core Group  Animal Disease Policy Group  Avian Expert Group  Avian Influenza  Animal and Plant Health Agency  Animal and Plant Health Inspection Services  Area Veterinarian in Charge  British Egg Industry Council  British Poultry Council  British Trust for Ornithology  Centre for Disease Control  Centre for Epidemiology and Animal Health  Chief Veterinary Officer  Department for Environment, Food and Rural Affairs  District Office  Environmental Protection Agency  Exotic and Emerging Animal Diseases  Foreign Animal Disease Diagnostician  Food and Agriculture Organisation of the United Nations  Foreign Agricultural Service  Food Standards Agency  Food Standards Scotland  Human Animal Infections and Risk Surveillance Group  Highly Pathogenic Avian Influenza  Health Protection Team  Low Pathogenic Avian Influenza  National Animal Health Laboratory Network  National Disease Control Centre  National Experts Group  National Emergency Epidemiology Group  National Health Service  National Incident Management Team  National Preparedness and Incident Coordination  National Poultry Improvement Plan  National Veterinary Services Laboratories  Outbreak Coordination Group  Ornithological Expert Panel  Outbreak Readiness Board  Regionalization Evaluation Services  Royal Society for the Protection of Birds  Royal Society for the Prevention of Cruelty to Animals  State Animal Health Official  Scottish Environmental Protection Agency  Statutory Nature Conservation Bodies  United Kingdom  UK Health Security Agency  UK Health and Safety Executive  United States of America  United States Animal Health Association  United States Department of Agriculture  Veterinary Exotic Notifiable Disease Unit  Veterinary Risk Group  Veterinary Services  World Health Organisation  World Organisation for Animal Health |
| --- | --- |
